# Perspectives on mental health amid the crisis of climate refugees

**DOI:** 10.47626/2237-6089-2024-0931

**Published:** 2025-05-26

**Authors:** Larissa Junkes, Antonio E. Nardi

**Affiliations:** 1 Universidade Federal do Rio de Janeiro Instituto de Psiquiatria Rio de Janeiro RJ Brazil Instituto de Psiquiatria (IPUB), Universidade Federal do Rio de Janeiro (UFRJ), Rio de Janeiro, RJ, Brazil.

## Introduction

Is there a connection between droughts in Panama, southern Africa, critical parts of the Amazon River, and the Syria, Iraq, and Iran region? What is the causal connection between extreme rainfall in East Africa, rainstorms in the UK, intense precipitation in the United Arab Emirates, and southern Brazil? Could the rising temperature of the Earth be responsible for deadly heatwaves across Asia, the extreme Sahel, and Madagascar? Scientists all over the world have been observing, with a mix of perplexity and frustration, the increasingly severe and deadly effects of the climate crisis, which has been underestimated for many years. Natural resources are being exploited only for the sake of progress, without regard for the environment, human lives, or physical and mental health, resulting in a slow and inadequate global response to disasters. There is a clear conflict between economic and political interests and the need to protect our planet and our lives. Actions are heading in the wrong direction, away from the necessary steps to slow down the current crisis. We all have two basic questions: is it still possible for us to come together as a collective to make a drastic change in this situation? Have we reached a tipping point where it is too late to turn back?

## The extreme case of Brazil and excessive rainfall worldwide

**Figure 1:**
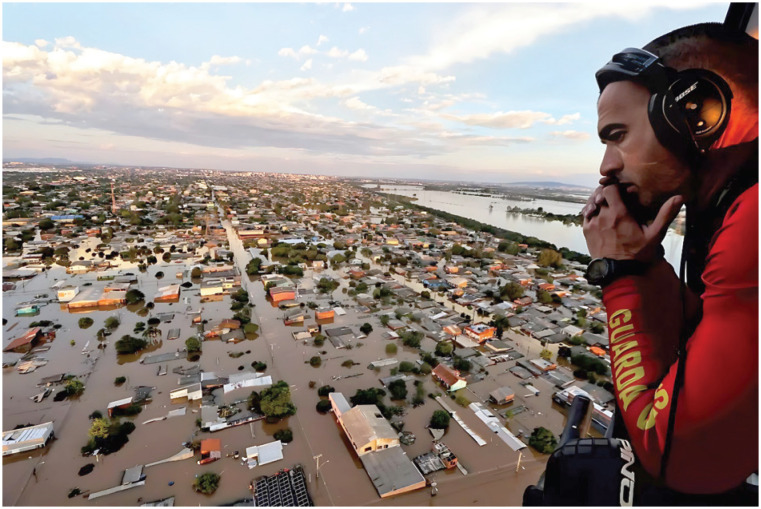
Rescue operation with the military fire department helicopter in the Metropolitan Region of Porto Alegre in May 2024. Photo: Lauro Alves/Secom, with permission.

In light of omissions, weakened environmental legislation, and numerous acts of climate crimes, we are witnessing an unprecedented increase in extreme events that are not accidental. Since the end of April 2024, the state of Rio Grande do Sul, in the southernmost part of Brazil, has been devastated by the largest flood in the region's history (Figure 1). This has resulted in more than 180 deaths and approximately 600,000 people being displaced, with over 80,000 housed in shelters.^1^ The state, which is larger than the United Kingdom in terms of territory, plays a significant role as an agricultural and industrial producer in Brazil, a country that is home to over 60% of the world's largest tropical forest, the Amazon Rainforest. What is the agenda to stop the colossal loss of biodiversity and the collapse of crucial ecosystems, including the Amazon? Shortly after the tragedy began in Rio Grande do Sul, in the northeastern region of Brazil, more than 30 municipalities in the state of Maranhão declared a state of emergency due to heavy rains.^2^

On the other side of the Atlantic, in May, devastating floods in East Africa caused widespread displacements, forcing hundreds of thousands of people to leave their homes in Burundi, Ethiopia, Kenya, Rwanda, Somalia, and Tanzania. Similarly, northern Afghanistan experienced sudden floods due to excessive seasonal rainfall. Before that, heavy rains caused floods and chaos in a desert region, Dubai. Additionally, Guangdong, a province in southern China with a population of 127 million, experienced record rainfall and extensive flooding. In many of these situations, marginalized groups living on the outskirts of the protective shield of the world's major economies are disproportionately affected by these disasters and are facing "invisible" injustices within the political-economic structure of inequality. All these events occurred within the first semester of 2024.

## The growing concern about climate refugees

What about the people affected by these disasters? Many lose their lives. Many others, who survive, face extremely traumatic situations such as the loss of loved ones and pets, the sudden abandonment of homes and livelihoods, and the need to seek shelter in unfamiliar locations away from their original community. According to data from the Civil Defense of Rio Grande do Sul, in June, more than 10,000 people were still living in shelters,^1^ some of which were housing units provided by the United Nations for refugees.^3^ The United Nations Refugee Agency (UNHCR) will implement international emergency responses that are tailored to the needs of Rio Grande do Sul, as they do around the world.

Victims also experience violence, misinformation, and uncertainty regarding the future. What lies ahead? A significant factor causing climate anxiety is the awareness that danger is looming, but not having appropriate skills to address it. This is known as eco-anxiety, which is the presence of anxiety related to the existential threat posed by ecological crises.^4^ While the priority immediately following a disaster is to save lives by moving people to safe locations, taking care of these survivors is rife with uncertainties. This applies in both the short term and in the long term as they try to reintegrate into a life that will never be the same anymore.

## Mental health is a public health issue

It is important to prioritize the physical and mental well-being of those affected by disasters, as they may struggle to act independently. The focus should be on providing safe care, relieving acute symptoms, restoring functionality, and preventing further suffering.^5^ It is crucial to approach these individuals without judgment and respect their privacy, looking out for warning signs of Acute Stress Disorder, Post-Traumatic Stress Disorder, panic attacks, feelings of hopelessness, depression, suicide risk, and aggression (see Table 1, based on TelePSI – Avaliação de Riscos em Saúde Mental^6^). In such extreme circumstances, the shortage of medications can pose an additional challenge.^7^ It is just as important to consider the mental health of caregivers involved in emergency response efforts, providing them with psychological support and personal care in an ethical and responsible manner.

**Table 1:** Approaches to mental health for climate refugees

**Overall assessment** Evaluation in a secure and private location Empathetic and attentive listening, without passing judgment Validation of suffering Respect for privacy and the right to make decisions Be mindful of the particular needs of children and teenagers	**What to assess** Primary concern Common changes: insomnia, anxious symptoms, sadness, anger, hypervigilance Previous psychiatric history and use of psychiatric medications General physical health conditions Basic needs: food, water, clothing, shelter, security, information
**Risk assessment**	**What to assess** Suicide Self and hetero-aggression Severe depression Psychosis Changes in critical judgment and insight, improper behavior, risk of being stigmatized
**Risk classification** Low: The patient presents the expected symptoms of suffering, which are consistent with the situation Alert: Intense and persistent suffering, signs of PTSD, depression, and decompensation of previous psychiatric illness Moderate: Patients experiencing chronic suffering or suicidal thoughts High: Patient experiencing acute distress and at risk of suicide	**How to conduct** Maintaining functionality, increasing resilience and preventing further suffering Refer to psychological and/or psychiatric assistance Refer to psychological and/or psychiatric assistance and make sure safety is ensured Refer to psychiatric and/or hospital assistance and ensure safety

Mental health professionals can help lessen the adverse effects of climate change on vulnerable populations by providing essential education and efficient care. This care involves appropriate responses to trauma resulting from climate-related disasters (see Table 2), utilizing approved mental health evaluations, and creating lasting therapeutic connections.^8^ The research on climate change and mental health is still in its early stages, but it is rapidly growing due to the complexity of the topic and the scarce data needed for evidence-based arguments. It can be challenging to pinpoint the direct impacts of climate crises on mental health and to establish a clear correlation between them.^9^

**Table 2:** Key considerations for mental health support during disasters

Performing the differential diagnosis between grief and depression amidst the crisis Patients experiencing sudden withdrawal of medications such as clozapine and anticonvulsants Symptoms of alcohol and drug withdrawal Attention to the accurate prescription of benzodiazepines

## Moving towards the past

It is important for us to recognize that climate change and extreme events may become permanent occurrences. The future outcomes of these events are uncertain, even if we were to stop deforestation and greenhouse gas emissions and completely shift from fossil fuels to renewable energy, a transition that is unfortunately unlikely to happen soon.^10,11^ The lack of progress on climate finance to help promote an equitable transition was disappointing at the twenty-eighth session of the Conference of Parties (COP28) on climate change in Dubai, United Arab Emirates, at the end of 2023.^12^ Mental health is becoming a fundamental issue, especially for populations residing in high-risk areas who may no longer be able to stay in their homes – in Brazil, over 2 million homes have been damaged or destroyed in extreme weather events between 2013 and 2022, resulting in a loss of approximately R$ 26 billion. This has affected 4,334 municipalities, which represents 78% of the country's municipalities, and has led to the displacement of more than 4.2 million people who either lost their homes or were forced to leave them.^13^ We are currently witnessing a series of forced migration movements that could escalate as a result of displacements caused by the climate crisis. This could potentially result in a resurgence of a form of ‘prehistoric’ nomadism where individuals had to continuously relocate in search of suitable living conditions, creating ‘temporary cities’. Investing in resilient cities is necessary. Urban areas need to be prepared for both current and future adversities to limit the magnitude and severity of the increasingly frequent environmental disasters.^14^ This serves as a clear reminder that we must take responsibility for our land and consider the well-being of future generations – which, ironically, includes ourselves. If we do not challenge the dysfunctional beliefs established by neoliberal policies aimed at short-term gains and take assertive action, there will be additional consequences affecting all of us.

There is a significant amount of work ahead as it is crucial to create resilient structures, now, as our current structures were designed for a climate that no longer exists, and we rely on systems that are woefully inadequate to keep us in safety. Can humans create structures that are as resilient as nature has been thus far? In order to survive climate change, we must adapt in ways that lead us towards a more inclusive and equal relationship with the environment, going against the plans of corporate expansion. The climate crisis is not a coincidence, and there is a dangerous complacency that is hindering our ability to achieve any sustainable balance. The responsibility for change is collective, to restore the natural balance and rebuild the bonds that we have systematically broken with our planet, in this ongoing destruction caused by unchecked consumerism born out of necessity. So far, we have not been able to confront the impending threat that is now upon us, thus climate collapse must become the new focus of public policies and infrastructure planning, all based on scientific evidence. Nothing will be resolved without deliberate action, just as we can no longer ignore the undeniable truth, even though it is important to continue to remind ourselves of it daily in the ongoing battle against misinformation. Worldwide, as in Brazil, promising currents in critical education are fostering our collective consciousness — underscoring the indispensable role of our public universities — and inspiring a more active engagement with collective processes. These processes increasingly call for coherent political decisions from our leaders, whose responses still too often echo with dissonance and fragmentation.

## The challenges of mental health in the context of climate crisis

The mental health crisis related to climate change is causing numerous concerns, prompting studies to explore the associations of multiple variables, including air pollution, heat exposure, loss of biodiversity, climate change, and mental health.^15-18^ There is a growing body of evidence showing how climate change significantly impacts mental health. Nations must urgently step up their efforts to address these changes, including mitigating the impact on mental health and psychosocial well-being.^19^ Nearly half of the global population and over 1 billion workers are exposed to episodes of intense heat, and about one third of all exposed workers experience negative health effects. Especially in tropical regions, the increase in warming may mean that physiological limits related to heat tolerance will be reached regularly and more frequently in the coming decades.^20^

According to data from the European Copernicus Climate Change Service, May 2024 marked 12 months of record global temperatures.^21^ In Europe, the average temperature for May 2024 was 0.88°C above the 1991-2020 average, making the month the third warmest May recorded for the continent. We need to reconnect deeply with the Earth by learning from the ancient teachings of indigenous peoples, recognizing and strengthening our unity as a planetary community, and addressing important differences to move beyond local efforts – such as those of indigenous communities protecting nature – towards solutions to global challenges. Leaders in planetary health, like the indigenous philosopher Ailton Krenak, an activist, urge us to reconsider our relationship with nature and view humanity as an integral part of it rather than separate. To fully embrace this concept as a society, it is crucial to enhance and broaden environmental policies, laws regarding environmental crimes, policies concerning solid waste, pesticides, and basic sanitation, water resource management, and ensuring the integrity in the creation and implementation of forest regulations.

Criticizing the notion of humans being separate from nature, Krenak advocates for a redefinition of our lives that aims to prevent the "end of the world."^22^ Indigenous territories have been at the forefront of resistance against the predatory exploitation of ecosystems – perhaps like few other population groups fully valuing nature before its collapse – and we need to be a part of this, within an integrated social perspective, with state economic policies supporting strategies that drive sustainable progress, setting aside proud arrogance. There is no other Earth, and we must keep our mental health support to its best.

## Data Availability

Not applicable.
